# Born at higher risk: persistent male excess risk of cerebral palsy from extreme prematurity to term: a systematic review and meta-analysis

**DOI:** 10.1186/s13293-026-00935-4

**Published:** 2026-06-01

**Authors:** Barbara Gardella, Mattia Dominoni, Alessia Arossa, Arsenio Spinillo

**Affiliations:** 1 Department of Clinical, Surgical, Diagnostic and Paediatric Sciences, University of Pavia, Pavia, 27100 Italy; 2 Department of Obstetrics and Gynecology, IRCCS Foundation Policlinico San Matteo, Pavia, 27100 Italy

**Keywords:** Cerebral palsy, Sex differences, Gestational age, Meta-analysis, Preterm birth, Neonatal outcomes

## Abstract

Male sex is recognized as a risk factor for cerebral palsy (CP), but the magnitude and consistency of this association across gestational ages remain unclear. To quantify sex-specific CP risk across gestational age categories and assess whether male excess risk varies by gestational age or temporal period, we conducted a systematic review and meta-analysis following PRISMA guidelines. Studies reporting sex-specific CP prevalence stratified by gestational age were included. Random-effects meta-analyses were performed stratified by gestational age (< 28 weeks, ≤ 31 weeks, 32–36 weeks, ≥ 37 weeks). Meta-regression assessed trends across gestational ages and temporal changes. Twenty-two studies comprising 47 comparisons, 15,734 CP cases and over 6.5 million births were included. Males showed consistently elevated CP risk across all gestational ages. Pooled risk ratios were: ≤31 weeks 1.27 (95% CI: 1.16–1.39, *p* < 0.001; I²=34.6%, high certainty), 32–36 weeks 1.12 (95% CI: 1.00-1.26, *p* = 0.05; I²=0.0%, moderate certainty), and ≥ 37 weeks 1.25 (95% CI: 1.18–1.32, *p* < 0.001; I²=37.8%, high certainty). Overall pooled RR was 1.22 (95% CI: 1.17–1.28, *p* < 0.001; I²=36.3%, high certainty). Meta-regression found no significant relationship between gestational age and male excess risk (β=-0.0007, *p* = 0.863, moderate certainty), indicating consistent male predominance across the gestational age spectrum. Finally, meta-regression, using span-weighted year of birth covariates (defined as the arithmetic mean of the first and last calendar year of the study cohort period, representing the temporal midpoint of birth exposure within each study and the study period duration, defined as the total span in years), showed that the male-to-female risk ratio for cerebral palsy in both preterm (< 37 weeks; 32 comparisons, omnibus Wald test = 5.07, *p* = 0.013, low certainty), very preterm (≤ 31 weeks; 24 comparisons, omnibus Wald test = 4.41, *p* = 0.02, moderate certainty) and extremely preterm (< 28 weeks, 7 comparisons, omnibus Wald test = 13.2,*p* = 0.0014,moderate certainty) infants is decreasing in more recent birth cohorts. Male sex confers 22–27% increased CP risk across all gestational ages. While this excess risk remains relatively constant across the gestational age spectrum, there is evidence of narrowing sex-specific differences among very preterm infants in recent years, possibly reflecting improvements in prenatal and neonatal care that differentially benefit males.

**Supplementary Information:**

The online version contains supplementary material available at 10.1186/s13293-026-00935-4.

## Introduction

Cerebral palsy (CP) represents the most common cause of physical disability in childhood, affecting approximately 2–3 per 1,000 live births worldwide [1–3]. While advances in obstetric and neonatal care have improved survival rates, particularly among preterm infants, the prevalence of CP has remained relatively stable. Understanding risk factors for CP is essential for developing targeted prevention strategies and improving outcomes for affected children and their families [3].

Male sex has long been recognized as a risk factor for various adverse neurodevelopmental outcomes, including autism spectrum disorders, attention-deficit/hyperactivity disorder, and intellectual disability [4–7]. Emerging evidence suggests that males are also at increased risk for CP since the 1980s, with most studies reporting male-to-female risk ratios between 1.2 and 1.4 [5, 7–8]. However, the consistency of this sex difference across different gestational ages and whether it has changed over time with improvements in perinatal care remain unclear.

Several biological mechanisms may contribute to increased male vulnerability to CP. Sex chromosomes themselves may play a role, with males lacking genetic redundancy for X-linked genes involved in neurodevelopment [6–8]. Hormonal factors, particularly androgens, have complex effects on brain development and may increase susceptibility to hypoxic-ischemic injury. Additionally, male fetuses and neonates demonstrate more robust pro-inflammatory responses to perinatal insults, which may exacerbate brain injury [7, 9–10]. Moreover, neonatal complications that are recognised antecedents of CP, including respiratory distress syndrome, intraventricular haemorrhage, sepsis, and necrotising enterocolitis, are consistently more prevalent and more severe in male than female preterm infants, independently of gestational age [6–7].

Gestational age at birth is one of the strongest predictors of CP risk, with extremely preterm infants facing substantially elevated risk compared to term-born infants [11]. However, whether the male excess risk for CP varies across gestational ages has not been systematically evaluated [8, 12–14]. If male vulnerability were primarily related to developmental immaturity, one might expect the sex difference to diminish with increasing gestational age. Conversely, if intrinsic sex-based biological factors predominate, the male excess risk might remain constant across the gestational age spectrum.

Furthermore, significant advances in neonatal care over recent decades, including increased use of antenatal corticosteroids and magnesium sulfate for neuroprotection, gentler ventilation strategies, and improved management of common neonatal complications, have transformed outcomes for preterm infants [2, 8, 14]. Whether these improvements have differentially benefited male versus female infants, potentially narrowing historical sex-based disparities in CP risk, remains unknown.

To address these knowledge gaps, we conducted a comprehensive systematic review and meta-analysis examining sex-specific CP risk across the full spectrum of gestational ages. Our objectives were to: (1) quantify the magnitude of male excess CP risk within specific gestational age categories; (2) determine whether this sex difference varies systematically with gestational age; and (3) assess temporal trends in sex-specific CP risk, particularly among very preterm infants who receive the most intensive neonatal care.

## Methods

### Methods

This systematic review and meta-analysis was registered prospectively in PROSPERO (CRD420251231478) (https://www.crd.york.ac.uk/PROSPERO/view/CRD420251231478) and was conducted according to PRISMA 2020 guidelines [15] (Supplement 1) and the Cochrane Handbook for Systematic Reviews of Interventions [16]. The protocol was developed prior to data extraction and analysis. The clinical question was defined according to the PICO framework: the **p**opulation comprised live-born infants across the full gestational age spectrum (extremely pretemr < 28 weeks, very preterm ≤ 31 weeks, moderate-to-late preterm 32–36 weeks, and term ≥ 37 weeks); the **e**xposure was male biological sex as recorded in birth registries or medical records; the **c**omparison was female sex within the same birth cohort; and the outcome was a diagnosis of cerebral palsy according to internationally accepted criteria, with the risk ratio (male versus female) as the primary effect measure.

### Search strategy

We systematically searched PubMed, Embase, Scopus, Web of Science, Cochrane Library, and CINAHL from inception to November 15, 2025, using the following terms: (“cerebral palsy” OR CP OR neurodevelopmental impairment) AND (sex OR gender OR male OR female) AND (“gestational age” OR preterm OR term OR “extreme prematurity”). MeSH terms included “Cerebral Palsy,” “Gestational Age,” and “Sex Characteristics.” No language or date restrictions were applied. The exact search strings used are available in PROSPERO (https://www.crd.york.ac.uk/PROSPEROFILES/12936808aacd3918f5e69f3f64e16bd6.pdf.) We included population-based cohort or registry studies reporting cerebral palsy cases stratified by both sex (male/female) and gestational age. Studies required ≥ 100 participants to ensure adequate statistical power. Randomized controlled trials and non-nested case-control studies were excluded. Two independent reviewers (AS, MD) extracted data using standardized forms, with discrepancies resolved by consensus. Variables included author, publication year, country, study period, total births and CP cases stratified by sex and gestational age, mean gestational age, and CP diagnostic criteria. Cases were defined as infants diagnosed with cerebral palsy according to standard criteria [1–2]. Studies were stratified into three primary gestational age categories: ≤31 weeks (very preterm), 32–36 weeks (moderate-to-late preterm), and ≥ 37 weeks (term). Secondary analyses compared preterm (< 37 weeks) versus term (≥ 37 weeks) births.

### Quality assessment

Two reviewers independently assessed study quality using the Newcastle-Ottawa Scale (NOS) for cohort studies [17]. This scale evaluates selection of cohorts (4 points), comparability of cohorts (2 points), and outcome assessment (3 points), with scores of ≥ 7 indicating high quality. Additionally, we evaluated the certainty of evidence using the Grading of Recommendations Assessment, Development and Evaluation (GRADE) approach for observational studies [18]. GRADE assessment considered risk of bias, inconsistency, indirectness, imprecision, and publication bias. (Supplement.2)

### Statistical analysis

The primary outcome was the risk ratio (RR) comparing CP risk in males versus females. We calculated study-specific RRs with 95% confidence intervals for each gestational age comparison. Random-effects meta-analysis using the DerSimonian-Laird method was performed to pool RRs, accounting for between-study heterogeneity.

Stratified meta-analyses were conducted for four gestational age categories: very preterm (≤ 31 weeks), moderate preterm (32–36 weeks), and term (≥ 37 weeks). Additional analyses grouped births as preterm (< 37 weeks) versus term (≥ 37 weeks). An overall meta-analysis pooling all gestational age strata was also performed. Secondary analysis (metaregression) included extremely preterm (< 28 weeks), very preterm and term categories.

Between-study heterogeneity was quantified using the I² statistic and τ² (tau-squared). I² values of 25%, 50%, and 75% were considered to represent low, moderate, and high heterogeneity, respectively. Cochran’s Q test assessed statistical significance of heterogeneity [16, 19]. To formally test whether gestational age modifies the magnitude of sex-specific risk, we conducted meta-regression with mean gestational age as a continuous predictor. To characterise temporal trends in sex-specific CP risk, we performed meta-regression analyses using two complementary study-level variables derived from the reported birth cohort period of each included study: (i) the midpoint year, defined as the arithmetic mean of the first and last year of the study period, and (ii) the study period duration, defined as the total span in years. Midpoint year captures the calendar-time position of average births (when neonatal care stood at the time); study duration captures temporal breadth (how many distinct care eras contributed births). Both variables were entered as continuous predictors in a joint model, allowing simultaneous estimation of the independent contribution of calendar time and study length to heterogeneity in the male excess CP risk. All models were fitted by restricted maximum likelihood (REML) [19]. The global contribution of the joint time-structure model was assessed via the omnibus Wald test (QM) (analogous to an F-test) [16, 19]. This approach has been recommended as preferable to using an arbitrary calendar threshold, because it avoids the assumption of a uniform distribution of births within the study period and extracts the full temporal information available from reported cohort intervals [16]. Publication bias was assessed using funnel plot visual inspection and Egger’s regression test and trim-and-fill analysis [16]. Sensitivity analyses included systematic leave-one-out analyses to assess the influence of individual studies on pooled estimates. All analyses were performed in R version 4.3.1 using the metafor package [19].

## Results

### Study selection and characteristics

**Fig. 1:**
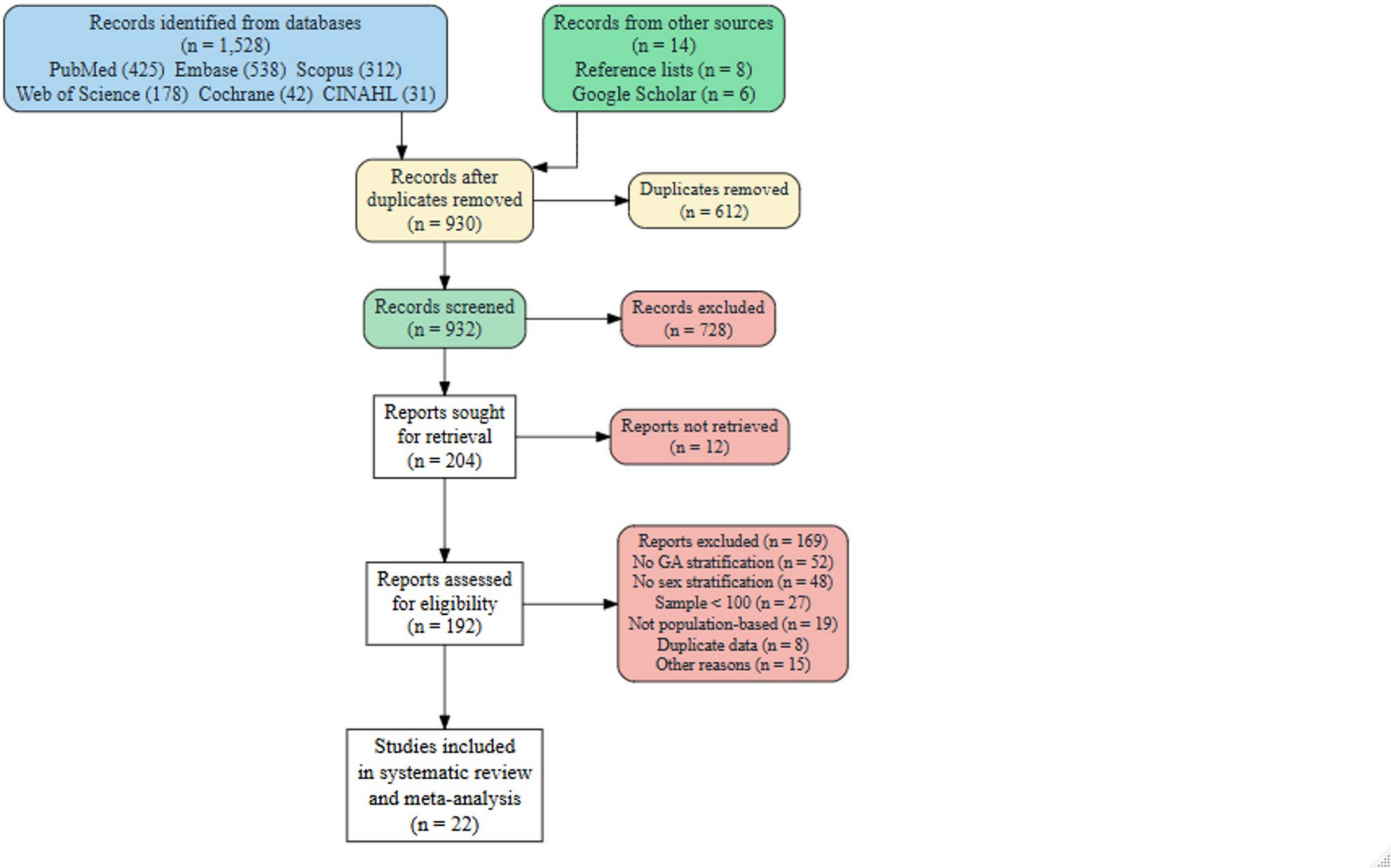
PRISMA flow diagram illustrating the study selection process

The systematic search identified 22 studies meeting inclusion criteria [8, 12–14, 20–37] providing 47 gestational age-specific comparisons encompassing 15734 CP cases and over 6.5 million births (Table 1). The PRISMA flow diagram of search and identification is presented in Fig. 1 and Suppl 1. Studies were published between 1997 and 2025 and originated from diverse geographic regions including Scandinavia (Sweden, Denmark, Norway, Finland), other European countries (France, United Kingdom, Austria), North America (USA, Canada), Asia (Japan, Korea), and Australia including births from 1967 to 2020. Four studies [21, 24, 32, 37] were published in the period 1997–2006, 10 studies [12, 13, 20, 22, 23, 27, 28, 30, 33, 35] in the period 2007–2016 and 8 [8, 14, 25, 26, 29, 31, 34, 36] in the period 2017–2025, respectively. All studies were from high-income countries with well-established perinatal care systems and no studies from low- or middle-income countries met inclusion criteria. None of the included studies provided both sex-specific mortality data and CP cases within the same population denominators. Consequently, CP risk could not be calculated relative to total live births, nor could competing risk due to differential mortality be formally addressed; pooled estimates therefore reflect CP risk among surviving infants at follow-up.

**Table 1:** Characteristics of included studies (*n* = 22). NOS scores indicate Newcastle-Ottawa scale quality assessment

Study	Country	Period	Sample size	GA categories	Data source	NOS score	Key findings	Comments
**Tran 2005²¹**	Australia	1989–1996	150	24-27w	Single center	**8/9**	Male sex interacted with maternal factors in predicting CP in extremely preterm infants	Small sample; single-center study limiting generalizability
**Hintz 2006** ^**32**^	USA (NICHD Network)	1997–2000	2,553 (1,337 F, 1,216 M)	< 28 weeks	NICHD NRN multicenter cohort	**8/9**	Boys had higher rates of moderate-severe CP (10.7% vs. 7.3%), MDI < 70 (41.9% vs. 27.1%), and NDI (48.1% vs. 34.1%). Male gender remained independent risk factor after adjustment (OR 2.0 for MDI < 70, OR 1.8 for NDI)	Multicenter prospective cohort with comprehensive Bayley assessment. Gender-specific multivariate analyses showed overlapping CIs for most risk factors. Limited by 62% follow-up rate
**Moore 2006²⁴**	UK	2006	584	22-26w	EPICure 2	**8/9**	Improved survival but persistent high CP rates in extremely preterm; male vulnerability noted	EPICure cohort; contemporary outcomes in UK
**Wu 2006** ^**37**^	USA (N. California, Kaiser)	1991–2002	334,333	≥ 36 weeks (term/near-term)	Kaiser Permanente population-based cohort	**8/9**	CP prevalence 1.1/1000. Neuroimaging findings: focal arterial infarction (22%), brain malformation (14%), periventricular white matter injury (12%). Risk factors: maternal age ≥ 35 (OR 1.9), black race (OR 1.4), IUGR <1st percentile (OR 4.3). IUGR most strongly associated with white matter injury (OR 17.5). 32% showed acute perinatal brain injury. No significant sex difference (54.7% male, RR 1.2, 95% CI 0.9–1.4, *p* = 0.16)	First US population-based CP study during neuroimaging era. 72% received neuroimaging (CT/MRI). Chart review by child neurologist. Demonstrates heterogeneous etiologies with different risk factor profiles by lesion type. Nighttime delivery associated with generalized atrophy but not other lesions. Population surveillance design; some families may have left system before diagnosis
**Johnson 2009²** **⁷**	UK	1995	219	23-25w	EPICure	**8/9**	High CP prevalence in extremely preterm survivors; male predominance observed	Original EPICure cohort; long-term follow-up data
**Beaino 2010** **¹²**	France	1997	1,812	24-32w	EPIPAGE cohort	**8/9**	Male excess in very preterm infants; gestational age and male sex independently predicted CP risk	EPIPAGE national cohort; comprehensive perinatal data. Follow-up: 89%
**Moster 2010** **¹³**	Norway	1967–2001	1,682,441	≥ 37w	Med Birth Registry	**9/9**	Male excess consistent across term gestational weeks (RR 1.19–1.61); largest population-based study	Complete national registry; long time span; high quality
**Kent 2012** ^**35**^	Australia (NSW/ACT)	1998–2004	2,549 (1,394 M, 1,155 F)	< 29 weeks (22-28w)	NSW/ACT NICU population-based registry	**8/9**	Hospital mortality higher in males (OR 1.285, 95% CI 1.035–1.595). Moderate/severe disability at 2–3 years more likely in males (OR 1.877, 95% CI 1.398–2.521). Males had more grade III/IV IVH, sepsis, major surgery. Gender differences lost significance at ≥ 27 weeks gestation	Population-based cohort from all 10 NICUs. Prospective data collection. Multivariate regression adjusted for multiple perinatal factors. Gender-specific analysis by gestational age subgroups. 75% follow-up rate at 2–3 years. Assessed with Griffiths/Bayley scales
**Neubauer 201** **2** ^**33**^	Austria (Tyrol)	2003–2008	377 (174 F, 203 M)	< 32 weeks	Population-based regional cohort	**8/9**	Girls had lower readmission rate in first year (28.7% vs. 49.8%, *p* < 0.0001), primarily due to fewer respiratory problems. No sex differences in neurodevelopmental outcomes (MDI, PDI, CP) at 12 or 24 months	Entire geographically defined population. High antenatal steroid use (~ 90%). After Bonferroni correction, minimal sex differences. Challenges conventional ‘male disadvantage’ hypothesis with modern care. Follow-up: 71%
**H** **irvonen 2014** **²⁸**	Finland	1991–2008	1,018,302	All GAs	National registries	**8/9**	Male excess across all gestational ages; highest risk in moderate-late preterm (32-36w)	Complete national data; comprehensive GA analysis
**Trønnes 2014³⁰**	Norway	1967–2001	62,572	24-36w	Med Birth Registry	**7/9**	Male sex increased CP risk in preterm births; interaction with pregnancy disorders examined	Subset of Moster 2010 focusing on preterm births
**Reid 2016** **²⁰**	Australia	1983–2009	1,663,557	All GAs	Victorian CP Register	**8/9**	Long-term population data confirming male sex as independent CP risk factor across all GAs	Comprehensive registry with clinical validation
**Serenius 2016²²**	Sweden	2004–2007	441	≤ 27w	EXPRESS	**8/9**	Comprehensive follow-up of extremely preterm showing male excess in neurodevelopmental outcomes	EXPRESS national cohort; detailed neurodevelopmental assessment. Follow-up: 94%
**Kuban 2016** ^**36**^	USA (ELGAN multicenter)	2002–2004 (10-yr f/u)	889 (428 F, 446 M)	< 28 weeks (23-27w)	ELGAN national multicenter cohort	**9/9**	At age 10, boys had higher prevalence of moderate/severe cognitive impairment (28% vs. 21%), microcephaly (15% vs. 8%, OR 2.4), motor impairment requiring devices (6% vs. 4%, OR 2.1), and ASD (9% vs. 5%, OR 2.0). No sex difference in seizure/epilepsy rates. Boy-to-girl ASD ratio (2:1) lower than general population (4:1)	Gold-standard neuropsychological assessment at 10 years (DAS-II, OWLS, NEPSY-II, ADI-R/ADOS-2). Excellent 92% follow-up rate. Adjusted for maternal and neonatal factors. Latent profile analysis identified 4 cognitive subgroups. Longest follow-up in contemporary EP cohort with comprehensive assessment. Boys 2x more likely to be severely impaired
**Pierrat 2017²²**	France	2011	2,714	24-31w	EPIPAGE-2	**8/9**	Contemporary cohort showing persistent male excess in neurodevelopmental impairment	Follow-up to EPIPAGE; shows persistent sex differences despite improved care
**Jöud 2020** **²⁶**	Sweden	1995–2014	215,217	All GAs	Skåne County	**8/9**	Male sex increased CP risk across gestational ages; interaction with other perinatal factors	Regional registry; interaction analyses with multiple risk factors. Follow-up: 94.5%
**Cortese 2021** ^**34**^	Norway	1967–2015	1,833,502	39-41w (term)	National Birth Registry	**9/9**	Birth weight < 3.5 kg associated with increased risk across all neurodevelopmental outcomes. Smallest babies had 25-fold risk for CP, 16-fold for vision/hearing disability, 11-fold for intellectual impairment. Patterns similar for boys and girls	Largest population-based study to date. Restricted to narrow GA range (39-41w) to eliminate prematurity confounding. Virtually complete follow-up via registry linkage
**Larsen 2022²** **⁵**	Denmark	1997–2017	995,498	All GAs	National registries	**8/9**	Week-specific analysis showed male RR ranging 1.04–1.61 across 27–31 weeks gestation	Detailed gestational-age-specific analysis; contemporary cohort
**Rouabhi 2023²⁹**	Canada	2003–2019	3,250	37-42w	Canadian CP Registry	**8/9**	Male sex included in predictive model for CP diagnosis in term infants	CP registry; prediction model development for term infants
**DeMauro 2024¹⁴**	USA	2008–2019	5,020	22-26w	NICHD NRN	**8/9**	Increasing CP prevalence in extremely preterm infants; RR 1.05 for males vs. females (95% CI: 0.96–1.16)	Most recent NICHD Network data; temporal trends analysis. Follow-up: 89.6%
**Haga 2025⁸**	Japan	2005–2020	666	< 30w	Single center	**8/9**	Male sex associated with higher CP risk (RR 1.82, 95% CI: 1.32–2.52) even without severe brain injury	Japanese cohort; documented male excess independent of brain injury. Follow-up: 82%
**Yang 2025³¹**	South Korea	2013–2019	1,829	23-31w	Korean Neonatal Network	**8/9**	Male sex associated with lower BSID-III scores and higher risk of neurodevelopmental delay in VLBW infants ≥ 26w GA. No sex differences in extremely preterm (23-25w). Cognitive/motor delay risk higher in 26-28w males; language delay risk higher in 29-31w males	Korean national network; GA-specific sex differences; most recent Asian data

The bold represents the significant value

### Meta-analysis by gestational age category

**Fig. 2:**
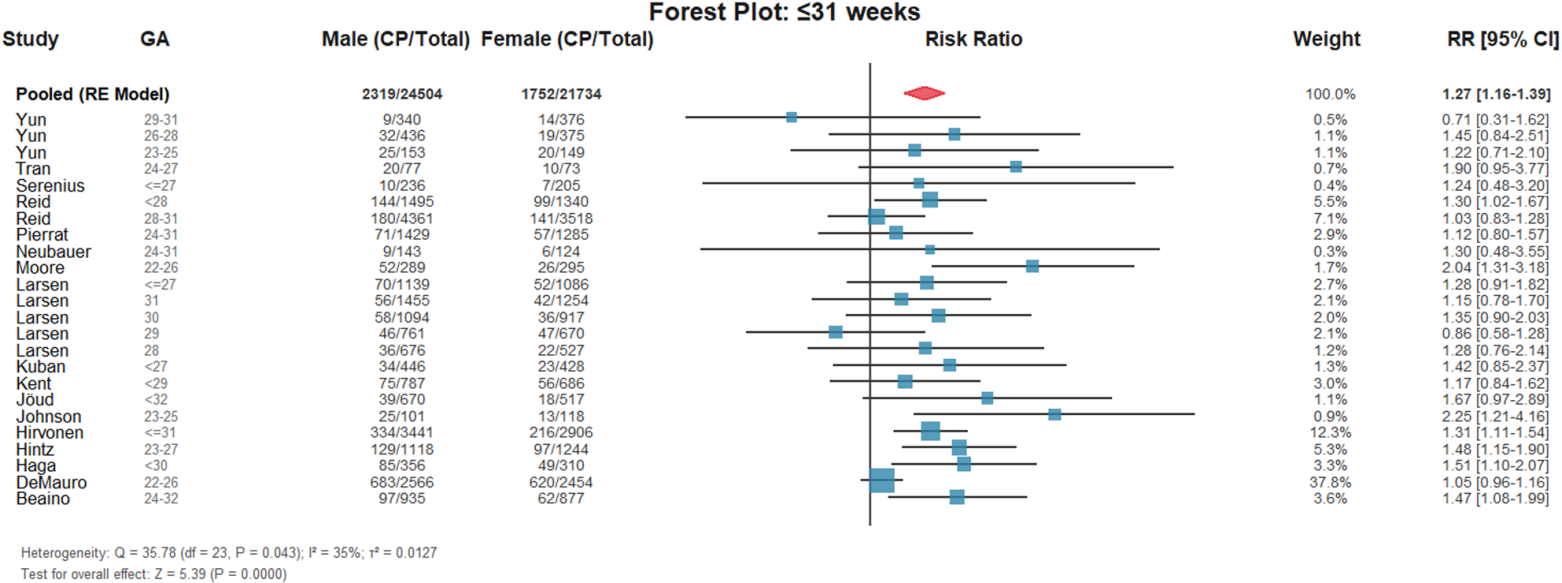
Forest plot of risk ratios for cerebral palsy comparing male to female infants born at ≤ 31 weeks’ gestation. The diamond represents the pooled estimate from random-effects meta-analysis.* Study of Beaino et al. included infants of 24–32 weeks of gestational age

**Very Preterm Births (≤ 31 weeks)**: Pooled analysis of 17 studies [8, 12, 14, 21–28, 30–33, 35–36] with 24 comparisons (46,238 total births) revealed a pooled risk ratio (RR) of 1.27 (95% CI: 1.16–1.39, *p* < 0.001, high certainty). Heterogeneity was moderate (I²=34.6%) (Fig. 2). Male prevalence was 94.64 per 1,000 births (95% CI: 91.00-98.37) compared to 80.61 per 1,000 (95% CI: 77.02–84.31) in females, representing an absolute risk difference of 14.03 per 1,000 births. Notable contributions came from large contemporary cohorts including the NICHD Neonatal Research Network study of 5,020 extremely preterm infants (22–26 weeks) [14], which showed an RR of 1.05 (0.96–1.16); the Japanese multicenter cohort of 666 infants (< 30 weeks) [8], reporting an RR of 1.82 (1.32–2.52); and the Danish national registry study [25] (995,498 births, gestational weeks 27–37) with 11 week-specific comparisons, showing RRs ranging from 1.04 to 1.61 across gestational ages from 27 to 31 weeks. In secondary analysis, in the 6 studies [14, 20, 21, 24, 27, 31] contributing to infants delivered before 28 weeks of pregnancy (9984 total births), the pooled risk ratio of CP among males compared to females was 1.42 (95% CI 1.13–1.79, *p* = 0.003, I^2^ = 65%).

**Fig. 3:**
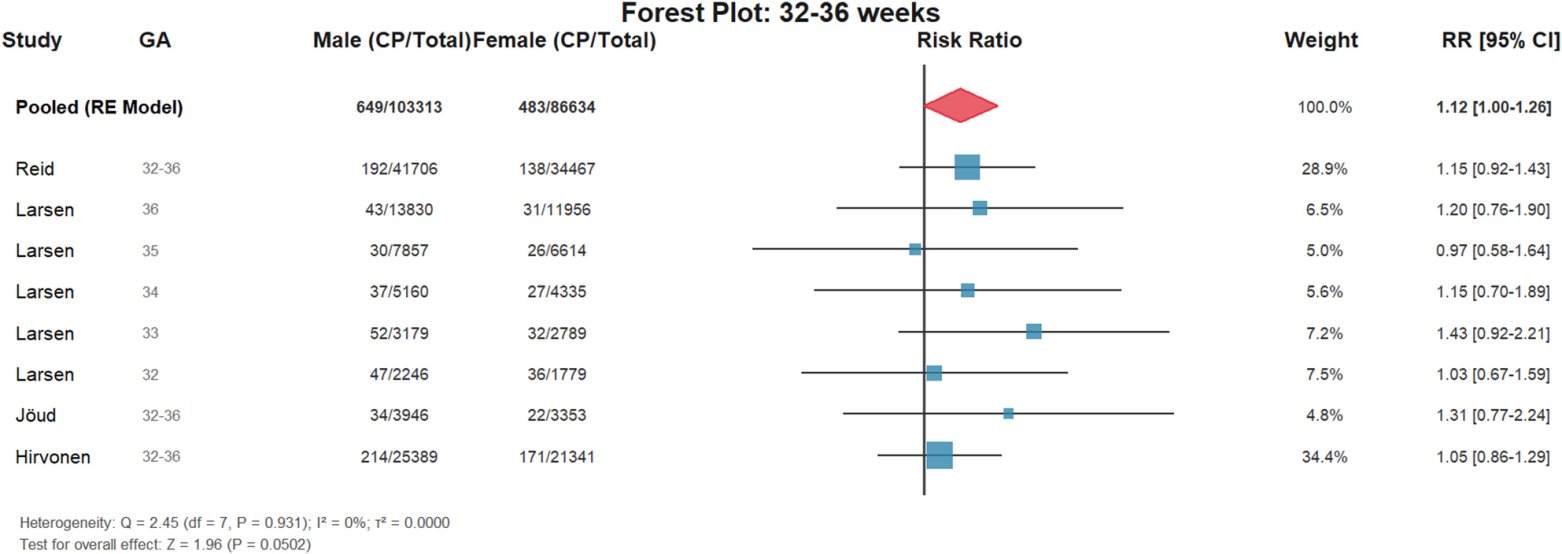
Forest plot of risk ratios for cerebral palsy comparing male to female infants born at 32–36 weeks’ gestation. The diamond represents the pooled estimate from random-effects meta-analysis

**Moderate Preterm Births (32–36 weeks)**: Among 8 comparisons from 4 studies [20, 24, 25, 27], the pooled risk ratio showed a trend toward male excess (pooled RR = 1.12, 95% CI: 1.00–1.26, *p* = 0.05, low certainty) (Fig. 3). Notably, heterogeneity was absent (I² = 0.0%), indicating remarkable consistency across studies from Australia, Denmark, Sweden, and Finland despite differences in study design, time periods, and population characteristics.The borderline statistical significance may reflect the lower baseline risk of CP in this gestational age range (9.1 per 1,000 in males, 7.9 per 1,000 in females) compared to earlier gestational ages, requiring larger sample sizes to detect sex differences. Combining all preterm births below 37 weeks, 17 studies with 32 comparisons yielded a pooled RR of 1.23 (95% CI 1.15–1.32, *p* < 0.001, I²=25.7%). (Suppl. 3 A). This combined preterm estimate differs from the stratum-specific analyses presented above in that it pools all gestational ages below 37 weeks.

The prevalence of preterm birth was 23.21 per 1,000 live births (95% CI: 22.39–24.05) in males versus 20.63 per 1,000 live births (95% CI: 19.80–21.50) in females.

**Fig. 4:**
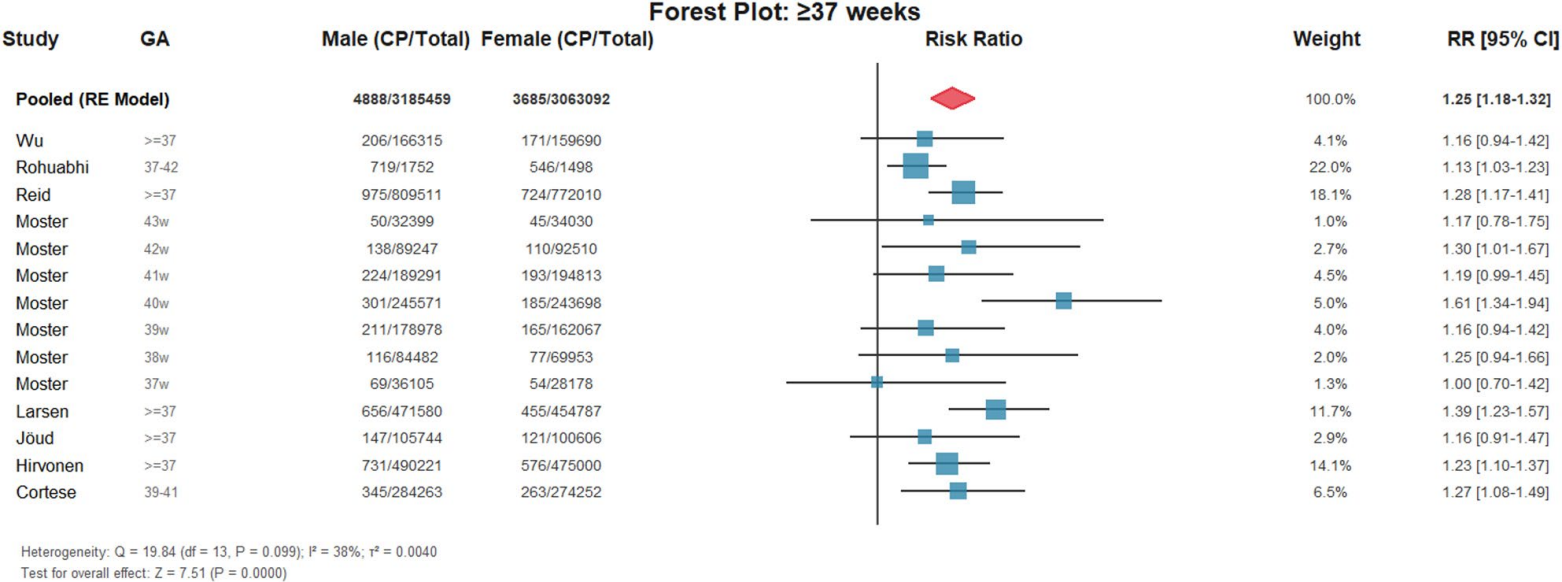
Forest plot of risk ratios for cerebral palsy comparing male to female infants born at ≥ 37 weeks’ gestation. The diamond represents the pooled estimate from random-effects meta-analysis

**Term Births (≥ 37 weeks)**: Analysis of 8 studies [13, 20, 25–26, 28–29, 34, 37] with 14 comparisons (6,248,551 total births) demonstrated a pooled RR of 1.25 (95% CI: 1.18–1.32, *p* < 0.001, high certainty). Heterogeneity was moderate (I²=37.8%). (Fig. 4, Suppl. 3 A). Male prevalence was 1.53 per 1,000 births (95% CI: 1.49–1.58) compared to 1.20 per 1,000 (95% CI: 1.16–1.24) in females, representing an absolute risk difference of 0.33 per 1,000 births. Meta-analysis pooling all 47 comparisons across all gestational ages yielded an overall RR of 1.22 (95% CI: 1.17–1.28, *p* < 0.001). Between-study heterogeneity was moderate (I²=36.3%).

### Meta-regression analysis

**Fig. 5:**
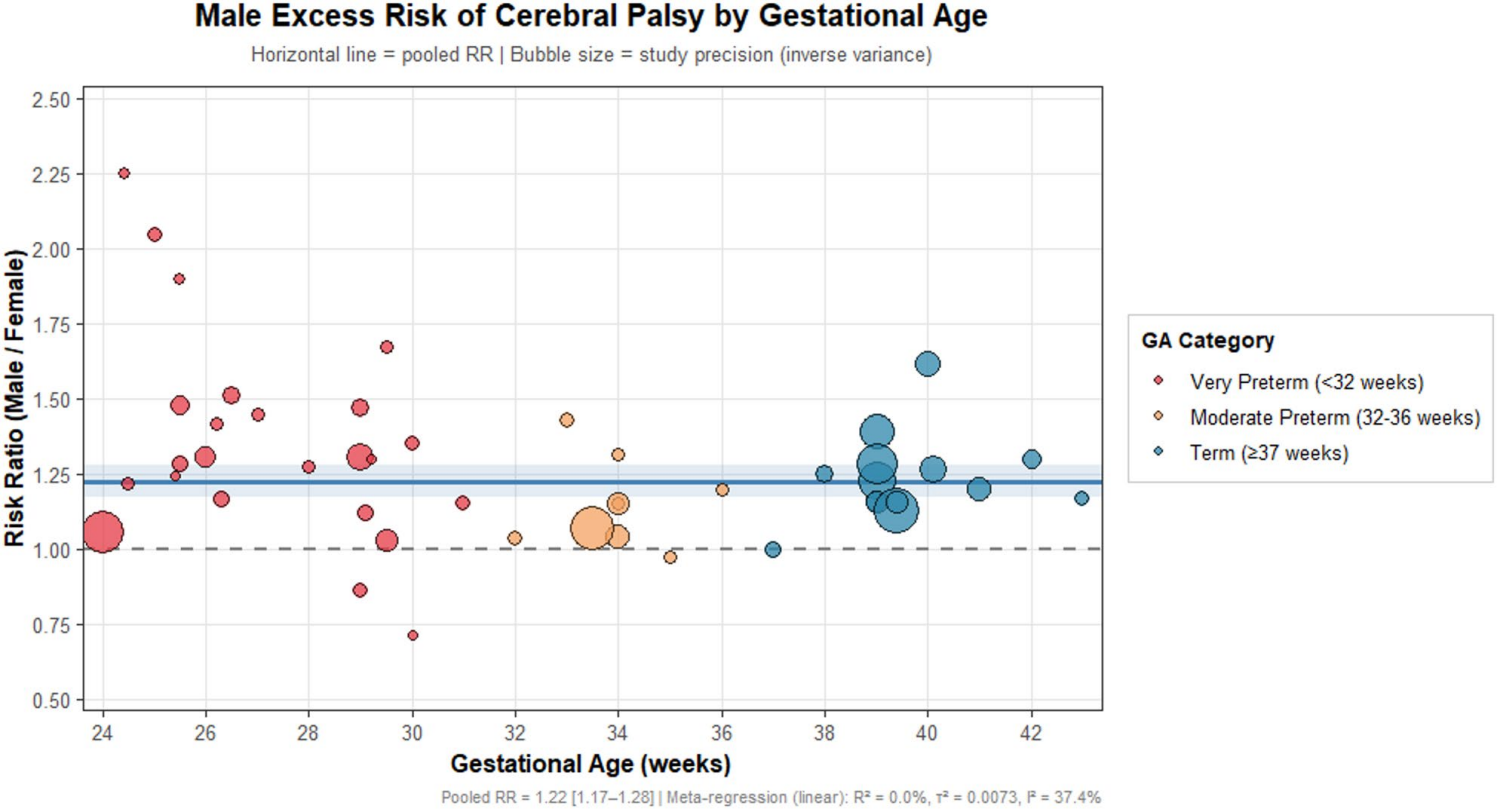
Meta-regression of log risk ratio (male vs. female) for cerebral palsy according to mean gestational age. Each circle represents one study comparison, with circle size proportional to study weight. The regression line shows no significant association between gestational age and the magnitude of male excess risk (β = 0.0013 (SE = 0.0045) *p* = 0.776)

To formally test whether gestational age modifies the magnitude of sex-specific risk, we conducted a meta-regression analysis with mean gestational age as a continuous predictor. Linear meta-regression found no significant relationship (β=-0.0007, standard error = 0.0041, *p* = 0.863, moderate certainty) between gestational age and RR of CP. (Fig. 5). Linear meta-regression models were also employed to assess changes in the male-to-female CP risk ratio over the time span of the cohorts. We employed a midpoint year variable to represent the specific era of neonatal care in which births occurred, alongside a study duration variable to account for temporal breadth and the range of clinical eras contributing to the dataset. Overall, across all 47 comparisons from 22 studies, the temporal correlation between male-to-female cerebral palsy (CP) risk, as constructed using midpoint and duration analyses of the cohorts, was not statistically significant according to the omnibus test (QM = 2.08, df = 3, *p* = 0.117; Suppl. 3B ). However, among preterm births (< 37 weeks) (32 comparisons), the joint model was statistically significant (QM = 5.07, df = 2, *p* = 0.013; pseudo-R² = 53.5%, Low certainty ), with cohort duration emerging as the dominant moderator across all inference methods (β = −0.01; cluster-robust *p* = 0.001). No significant time effect was detected in term births (≥ 37 weeks, 14 comparisons; QM *p* = 0.678, pseudo-R² = 0%; Suppl. 3B). Additionally, among the 24 comparisons restricted to very preterm births (≤ 31 weeks of gestation), the joint model was statistically significant (QM = 4.41, df = 2, *p* = 0.025, midpoint year and duration analysis robust ep values < 0.001, moderate certainty) and explained 59.1% of the between-study variance, indicating a significant moderating signal across all analyses (Suppl. 3 C) In addition, collinearity between the two predictors in the ≤ 31-week stratum is negligible (*r* = − 0.091) indicating that the two predictors were non-redundant. The joint model for extremely preterm infants (< 28 weeks; 7 comparisons) was significant (QM = 13.2, *p* = 0.0014; moderate certainty). With robust p-values of 0.015 for midpoint and 0.04 for duration, and high explained variance (R² = 94.4%), the results indicate a significant reduction in the male excess risk of CP over time within this category. We also performed an era-stratified analysis classifying comparisons by birth-cohort midpoint year into three eras. For ≤ 31-week births, pooled RRs declined monotonically: in the pre-2000 era (k = 7 comparisons, 6 studies), pooled RR = 1.34 (95% CI 1.17–1.54, I²=41.6%); in the 2000–2010 era (k = 11, 7 studies), RR = 1.28 (95% CI 1.12–1.46, I²=0%); in the post-2010 era (k = 6, 4 studies), RR = 1.15 (95% CI 0.99–1.35, *p* = 0.070, I²=26.7%) (Suppl. 3 C). The post-2010 estimate crosses the conventional significance threshold for the first time. For ≥ 37-week term births, pooled RRs were numerically stable across eras. No eligible study was available in the post-2010 era for the 32–36 week stratum. .

### Publication bias and sensitivity analyses

Comprehensive publication bias assessment was conducted using multiple complementary approaches (Supplement 3E-F).Egger’s regression test for funnel plot asymmetry yielded z = 1.82, *p* = 0.076, suggesting no significant small-study effects. However, the p-value approaching significance warranted additional investigation. Trim-and-fill analysis identified potential asymmetry, imputing 8 missing studies on the left side of the funnel plot (Supplement 3). The adjusted pooled RR was 1.18 (95% CI: 1.12–1.24), representing a modest 3.6% reduction from the original estimate of 1.22. Influence diagnostics and leave-one-out analyses identified no single study exerting disproportionate influence on pooled estimates. Sequential omission of individual studies produced minimal variation in effect sizes, with all leave-one-out estimates remaining within 10% of the original pooled RR (Supplement 3 F).

### Quality assessment and certainty of evidence

Quality assessment using the Newcastle-Ottawa Scale showed that all 22 included studies achieved moderate to high quality scores. Detailed quality assessment results are reported in Suppl. 2. All studies employed population-based cohort designs or used established CP registries, ensuring representative ascertainment of cases. The majority of studies utilized standardized CP diagnostic criteria based on internationally accepted definitions (e.g., Surveillance of Cerebral Palsy in Europe criteria or similar frameworks). Follow-up adequacy was satisfactory in all studies, with CP assessment typically conducted at ages 2–5 years when diagnosis is most reliable.

The certainty of evidence was assessed using the GRADE framework, with all observational analyses starting at low certainty. The overall pooled estimate of male CP risk was rated high certainty, based on two concurrent upgrade criteria: consistent direction across all 47 comparisons from 22 independent populations with no stratum crossing the null. Heterogeneity was moderate (I² = 35.8%) but without directional inconsistency, and no evidence of publication bias. For very preterm births (≤ 31 weeks), the certainty was rated as high. The evidence was upgraded by two points because: (1) all studies were high-quality population-based cohorts; (2) the large effect size (RR = 1.27) was consistent across studies; (3) heterogeneity was moderate (I²=34.6%) but explained by temporal trends; (4) no serious risk of bias or publication bias was detected; and (5) the absolute effect was clinically important (14 additional CP cases per 1,000 male births). For moderate-to-late preterm births (32–36 weeks), the certainty was rated as low. Lack of upgrade was due to : (1) lower precision, with the 95% confidence interval including next to null value (RR 1.12, 95% CI: 1.00-1.26); and (2) smaller absolute risk difference (0.7 additional cases per 1,000 male births), making the clinical importance less certain. For term births (≥ 37 weeks), the certainty was judged as high. The evidence was upgraded because: (1) the estimate was highly precise (RR = 1.25, 95% CI: 1.18–1.32); (2) the direction of effect was consistent across all studies; (3) the very large underlying population (> 6 million term births) provided excellent statistical power; (4) heterogeneity was moderate (I²=37.8%) but did not obscure the consistent direction of effect; and (5) high-quality study designs with validated CP registries. For the overall finding of consistency across gestational ages (meta-regression analysis), the certainty was rated as moderate. This rating was based on: (1) robust meta-regression methodology showing no significant gestational age interaction (*p* = 0.863); (2) consistency of male excess risk across 47 independent comparisons spanning extreme prematurity to term; (3) biological plausibility supported by extensive mechanistic research. (4) one downgrade for the ecological nature and limited study-level observations of the interaction test. For the joint temporal meta-regression, certainty was rated low in the full preterm stratum reflecting one upgrade for the internal concordance and robustness of both temporal variables but two downgrade steps for ecological risk of bias and indirectness. In the very preterm and extremely preterm strata, certainty was upgraded to moderate by the GRADE dose-response gradient criterion: the temporal moderation was strongest in the most NICU-dependent infants, present in the broader preterm stratum, and completely absent at term, precisely the biological gradient predicted if progressive improvements in neonatal intensive care have disproportionately narrowed the male-specific CP disadvantage in the most immature newborns.

## Discussion

### Summary of principal findings

In this systematic review and meta-analysis of over 6.5 million births from 22 cohorts, we found a consistent male excess risk of cerebral palsy (CP) across the entire gestational age spectrum. Male infants had a significantly higher risk of CP in very preterm (≤ 31 weeks), moderate preterm (32–36 weeks) and term births (≥ 37 weeks). Importantly, meta-regression detected no significant interaction between gestational age and fetal sex, indicating that the magnitude of male excess was remarkably stable despite the large differences in absolute CP prevalence across gestational ages. The stability of the male excess across gestational ages suggests that this disparity is not solely attributable to prematurity-related morbidities but reflects intrinsic sex-based neurobiological vulnerability ^7^. We identified a significant overall temporal reduction in male excess risk among very preterm infants, with no change in moderate-late preterm births and term births. This suggests that substantial improvement of perinatal care in the last decades has reduced the CP risk gap between males and females among premature infants.

### Comparison with previous literature

The male-to-female ratio of 1.2–1.3:1 identified in our meta-analysis is remarkably consistent with reports from individual population-based registries. Male excess across different CP subtypes and severity levels has been reported across Swedish birth cohorts spanning 1999–2010 [38]. The Surveillance of Cerebral Palsy in Europe (SCPE) collaboration, which aggregates data from multiple European registries, has consistently reported male predominance of similar magnitude [39]. Population-based studies from Scandinavia [13], Australia [20], and the UK [23] similarly report a stable male predominance among children with CP. However, none of these syntheses stratified the sex effect by gestational age, so the question of whether male vulnerability is amplified at the extremes of viability remained unresolved. In a large study of the Swedish population, Chen et al. [40] found that the proportion of CP risk mediated by preterm neonatal morbidities (respiratory distress, bronchopulmonary dysplasia) is exceptionally high at the lowest gestational ages (91.7% at 24 weeks) and decreases as gestation advances, with the leading mediators shifting from asphyxia in the early preterm period to neurological-related diseases (such as intracranial hemorrhage and periventricular leukomalacia) from 34 weeks onwards. The rates of prematurity-related complications are consistently higher in male than in female neonates [41–42], thus confirming that male sex could add to the overwhelming effect of neonatal morbidities on the risk of subsequent cerebral palsy among premature infants. The persistence of male CP excess even in term births suggests that the neurological vulnerabilities conferring increased male risk may be less dependent on gestational maturity than are respiratory vulnerabilities [28, 41–43]. The absence of a gestational age gradient in male excess risk is a striking finding of our study. It suggests that the male predisposition is an intrinsic factor that heightens susceptibility to brain injury from a wide array of insults, whether they are the asphyxia-dominated injuries of extreme prematurity or the neurological injuries more common in late preterm and term infants.

### Biological mechanisms

The biological mechanisms causing the excess risk of brain lesions among male infants, or protection of females, involve sex hormone-mediated mechanisms of apoptosis or differential vulnerability to perinatal asphyxia, which are particularly effective in preterm infants [44]. Conversely, relative female resistance may derive from neuroprotective mechanisms, such as estrogen-modulated anti-apoptotic pathways and XX-linked redundancy, which appear to operate consistently throughout gestation [7, 44]. Other mechanisms include a dimorphic neuronal sexual response to inflammatory and oxidative stress stimuli potentially mediating brain damage after infection or asphyxia [43]. Whatever the mediators of cerebral palsy in different gestational ages, the male excess risk remains almost uniform, suggesting an inherent male vulnerability that operates independently of the intermediate cause (respiratory problems, asphyxia, infection, inflammation). The male excess in cerebral palsy is not a fixed phenomenon but has decreased over time, as demonstrated by a significant inverse temporal relationship between male-to-female risk ratio among very preterm infants. This decline has occurred in parallel with improvements in perinatal and neonatal care. These improvements correspond closely with the widespread adoption of evidence-based neonatal intensive care unit (NICU) interventions, including surfactant therapy, antenatal corticosteroids, gentler ventilation strategies, and enhanced infection prevention measures. These interventions have had a particularly beneficial effect on male infants, who are known to be more vulnerable than female infants to several key complications of preterm birth, notably respiratory distress syndrome and sepsis [41–44]. While sex-specific biological differences, such as slower lung maturation, heightened inflammatory responses, and sex-dimorphic brain development [40–42], establish a baseline male predisposition to vulnerability, the phenotypic expression of this risk is modifiable. Advances in neonatal care, particularly the introduction of surfactant therapy, antenatal corticosteroids, and gentler ventilation strategies, appear to have attenuated (though not eliminated) the male excess in adverse outcomes. This suggests that the clinical consequences of these inherent biological differences can be meaningfully mitigated, even when the underlying sex-based differences themselves persist. However, the extent to which these improvements can further narrow or potentially eliminate the remaining male disadvantage remains uncertain.

### Strengths and Limitations

The main strengths of this meta-analysis include a large sample size and geographic diversity of populations included. The inclusion of birth cohorts spanning five decades (1967–2020) allowed examination of whether sex differences have changed over time alongside dramatic improvements in perinatal care. The majority of included studies were based on well-established CP registries with standardized diagnostic criteria, ensuring methodological consistency. Finally, by examining sex ratios across gestational age categories, we were able to identify patterns that would have been obscured in analyses treating all CP cases as a homogeneous group. Main limitations include potential heterogeneity between studies due to differences in included populations, different criteria used to diagnose cerebral palsy during different decades, and different follow-up times. Residual confounding from factors differentially distributed between sexes, including fetal growth restriction, congenital anomalies, and multiple gestation, cannot be formally excluded without individual-patient data. Most studies did not report data by CP subtype, precluding subtype-specific analysis. Finally, moderate heterogeneity in some categories suggests unmeasured sources of between-study variation, though temporal factors explained heterogeneity in very preterm births. In addition, most of the available data come from high-income countries with well-established CP registries, predominantly in Europe and Scandinavia. Whether similar sex differences exist in populations with different genetics, maternal nutrition, infectious disease burdens, or access to perinatal care remains uncertain. Most included studies reported CP prevalence among survivors rather than per live birth. Because male infants, particularly those born preterm, experience higher neonatal mortality [33, 36, 42], differential survival could influence observed sex-specific CP risk through competing risk mechanisms. If more vulnerable males die before CP diagnosis, the male excess among survivors may underestimate the true biological vulnerability. However, the persistence of male excess across gestational ages, including term births where mortality is minimal, suggests that differential survival alone is unlikely to account for our findings. Our requirement for sex-stratified, gestational-age-specific data led to the exclusion of 48 otherwise eligible studies. While this could theoretically introduce selection bias, if studies finding no sex difference were less likely to stratify by sex, several factors mitigate this concern. Trim-and-fill analysis detected no evidence of publication bias, and the pooled estimates remained consistent across 47 comparisons from 22 studies conducted in diverse populations and time periods. Moreover, many of the excluded studies were large registries that did not stratify by sex for operational rather than analytical reasons. Finally, the findings of a reduction of male-to-female CP risk in recent cohorts should be regarded as biologically plausible and mechanistically coherent, but requires confirmation in individual-patient data analyses before informing clinical recommendations.

## Conclusions

This systematic review and meta-analysis provides robust evidence of a consistent male excess in cerebral palsy risk, with males comprising approximately 56–58% of cerebral palsy cases across all gestational ages. This sex difference in cerebral palsy risk remains stable across gestational age spectrum. However, the magnitude of this male excess has decreased over time among preterm and very preterm infants. The persistence of a consistent sex difference across all gestational ages, despite the observed reduction in the magnitude of male excess over time, suggests that fundamental biological mechanisms are primarily responsible for the male vulnerability to cerebral palsy. The temporal decrease in the male excess, particularly among very preterm infants, indicates that improvements in perinatal care have reduced the disparity in cerebral palsy risk between male and female infants.

## Supplementary Information

Below is the link to the electronic supplementary material.

Supplementary Material 1: PRISMA 2020 Checklist.

Supplementary Material 2: Detailed Newcastle-Ottawa Scale quality assessment for each included study. Table of grade assignment.

Supplementary Material 3: A.Overall (preterm vs term) analysis. B. Funnel plot publication analysis with sensitivy analysis (trim-and-fill). C. leave-one-out analysis. D: Male excess risk according to the proportion of infants born last 25 years stratified by gestational age. Overall gestational age analysis: linear term β=-0.0007 (p=0.863). Very preterm births (≤31 weeks) linear term β=-0.0023 (SE=0.00086, p=0.007, 32-36:β=0.0008, p value= 0.934, ≥37:β=0.0003,p value= 0.6.

## Data Availability

No datasets were generated or analysed during the current study.

## Abbreviations

CP: Cerebral palsy
NOS: Newcastle-Ottawa Scale
ROB: Risk of bias
RR: Relative risk
95% CI: 95% Confidence Interval
EPT: Extremely preterm birth (< 28 weeks)
VPT: Very prterm birth (28–31 weeks)
