# Supplementary material for: Born at higher risk: persistent male excess risk of cerebral palsy from extreme prematurity to term: a systematic review and meta-analysis
Source: Biol Sex Differ. 2026 Jun 1;17:143. doi: 10.1186/s13293-026-00935-4 (PMC13445702; doi:10.1186/s13293-026-00935-4)
Supplement: Supplementary file 2 [file 13293_2026_935_MOESM2_ESM.docx]

Detailed quality assessment of all included studies using the Newcastle-Ottawa Scale (NOS) for cohort studies.

## Tran et al. 2005 (Ref 21)

**Total NOS Score: 8/9** (Selection: 4/4, Comparability: 2/2, Outcome: 2/3)

- • Selection: Single tertiary center; clear case definition; matched controls
- • Comparability: Matched on GA; controlled for multiple factors
- • Outcome: Validated CP diagnosis; adequate follow-up; high response rate

## Hintz et al. 2006 (Ref 32)

**Total NOS Score: 8/9** (Selection: 4/4, Comparability: 2/2, Outcome: 2/3)

- • Selection: Representative national cohort (NICHD NRN), secure record, outcome not present at baseline
- • Comparability: Controls for gestational age, IUGR, multiple neonatal morbidities
- • Outcome: Validated assessment by Bayley-II; adequate follow-up; 62% follow-up rate

## Moore et al. 2006 (Ref 24)

**Total NOS Score: 8/9** (Selection: 4/4, Comparability: 2/2, Outcome: 2/3)

- • Selection: National population-based (EPICure 2); prospective design
- • Comparability: Controlled for multiple perinatal and socioeconomic factors
- • Outcome: Standardized neurologic assessment; adequate follow-up duration; good follow-up rate

## Wu et al. 2006 (Ref 37)

**Total NOS Score: 8/9** (Selection: 4/4, Comparability: 2/2, Outcome: 2/3)

- • Selection: Representative population-based cohort (Kaiser Permanente); electronic records validated by chart review
- • Comparability: Adjusted for maternal age, ethnicity, plurality, and IUGR with polytomous regression by neuroimaging type
- • Outcome: CP diagnosis by child neurologist; 72% received neuroimaging; population surveillance design

## Johnson et al. 2009 (Ref 27)

**Total NOS Score: 8/9** (Selection: 4/4, Comparability: 1/2, Outcome: 3/3)

- • Selection: National population-based (EPICure); prospective design
- • Comparability: Controlled for socioeconomic status; limited control for other confounders
- • Outcome: Direct standardized assessments (K-ABC, GMFCS); 11-year follow-up; 71% follow-up with imputation

## Beaino et al. 2010 (Ref 12)

**Total NOS Score: 8/9** (Selection: 4/4, Comparability: 2/2, Outcome: 2/3)

- • Selection: Representative national cohort (EPIPAGE), secure record, outcome not present at baseline
- • Comparability: Controls for gestational age and multiple neonatal morbidities
- • Outcome: Validated CP diagnosis by specialists; adequate follow-up (89%); follow-up to 5 years

## Moster et al. 2010 (Ref 13)

**Total NOS Score: 9/9** (Selection: 4/4, Comparability: 2/2, Outcome: 3/3)

- • Selection: Complete Norwegian national birth cohort; secure medical birth registry
- • Comparability: Comprehensive adjustment for maternal, pregnancy, and delivery factors
- • Outcome: Validated CP diagnosis; long-term follow-up (up to 39 years); complete registry follow-up

## Kent et al. 2012 (Ref 35)

**Total NOS Score: 8/9** (Selection: 4/4, Comparability: 2/2, Outcome: 2/3)

- • Selection: Population-based cohort (all NSW/ACT NICUs); prospective registry; secure records
- • Comparability: Adjusted for GA, SGA, multiple perinatal factors with stratified analysis by GA groups
- • Outcome: Standardized assessment (Griffiths/Bayley); adequate follow-up; 75% follow-up rate

## Neubauer et al. 2012 (Ref 33)

**Total NOS Score: 8/9** (Selection: 4/4, Comparability: 2/2, Outcome: 2/3)

- • Selection: Complete geographically defined population (Tyrol); prospective cohort; secure records
- • Comparability: Adjusted for GA, birth weight, maternal factors; sibling analysis; Bonferroni correction
- • Outcome: Standardized Bayley assessment at 12 and 24 months; 71% follow-up rate

## Hirvonen et al. 2014 (Ref 28)

**Total NOS Score: 8/9** (Selection: 4/4, Comparability: 2/2, Outcome: 2/3)

- • Selection: Complete Finnish national birth cohort; secure national registries
- • Comparability: Stratified by GA; adjusted for multiple confounders
- • Outcome: Registry-based CP diagnosis; follow-up to 7 years; population-based follow-up

## Trønnes et al. 2014 (Ref 30)

**Total NOS Score: 7/9** (Selection: 4/4, Comparability: 2/2, Outcome: 1/3)

- • Selection: Complete Norwegian national cohort; secure registries
- • Comparability: Adjusted for multiple pregnancy and sociodemographic factors
- • Outcome: Registry-based diagnosis (potential under-ascertainment of mild cases); long follow-up; some missing data

## Reid et al. 2016 (Ref 20)

**Total NOS Score: 8/9** (Selection: 4/4, Comparability: 2/2, Outcome: 2/3)

- • Selection: Complete Victorian population; validated CP register
- • Comparability: Adjusted for GA, birth weight, and multiple factors
- • Outcome: Validated register with specialist confirmation; complete population follow-up

## Serenius et al. 2016 (Ref 22)

**Total NOS Score: 8/9** (Selection: 4/4, Comparability: 2/2, Outcome: 2/3)

- • Selection: National Swedish cohort (EXPRESS); prospective design
- • Comparability: Adjusted for GA, maternal education, country of birth
- • Outcome: Standardized assessment at 2.5 years; 94% follow-up for preterm group

## Kuban et al. 2016 (Ref 36)

**Total NOS Score: 9/9** (Selection: 4/4, Comparability: 2/2, Outcome: 3/3)

- • Selection: Representative national cohort (ELGAN); prospective enrollment; validated procedures; outcome assessed at 10 years
- • Comparability: Comprehensive multivariable adjustment with multinomial regression; latent profile analysis
- • Outcome: Gold-standard comprehensive battery (DAS-II, OWLS, NEPSY-II, ADI-R/ADOS-2); 10-year follow-up; 92% follow-up rate

## Pierrat et al. 2017 (Ref 23)

**Total NOS Score: 8/9** (Selection: 4/4, Comparability: 2/2, Outcome: 2/3)

- • Selection: National cohort (EPIPAGE-2); prospective design; secure records
- • Comparability: Adjusted for multiple perinatal and neonatal factors
- • Outcome: Standardized 2-year assessment; high follow-up rate (88%)

## Jöud et al. 2020 (Ref 26)

**Total NOS Score: 8/9** (Selection: 4/4, Comparability: 1/2, Outcome: 3/3)

- • Selection: Complete regional cohort (Skåne County); secure birth registry
- • Comparability: Stratified by GA; univariate analyses within strata
- • Outcome: Validated CP register (CPUP); follow-up to 2+ years; 94.5% follow-up

## Cortese et al. 2021 (Ref 34)

**Total NOS Score: 9/9** (Selection: 4/4, Comparability: 2/2, Outcome: 3/3)

- • Selection: Complete national population (Norwegian Medical Birth Registry); validated registry linkage; prospective outcomes
- • Comparability: Comprehensive adjustment for maternal and infant factors; sibling analysis to control familial confounding
- • Outcome: Multiple validated neurodevelopmental outcomes; extended follow-up (up to 47 years); virtually complete registry follow-up

## Larsen et al. 2022 (Ref 25)

**Total NOS Score: 8/9** (Selection: 4/4, Comparability: 1/2, Outcome: 3/3)

- • Selection: Complete Danish national cohort; secure medical birth register
- • Comparability: Adjusted for sex; limited control for SES and other factors
- • Outcome: Validated national CP register; follow-up to 6 years; complete registry follow-up

## Rouabhi et al. 2023 (Ref 29)

**Total NOS Score: 8/9** (Selection: 4/4, Comparability: 2/2, Outcome: 2/3)

- • Selection: National Canadian CP Registry; validated diagnoses
- • Comparability: Controlled for multiple perinatal and socioeconomic factors
- • Outcome: Specialist-confirmed CP diagnosis; standardized data collection

## DeMauro et al. 2024 (Ref 14)

**Total NOS Score: 8/9** (Selection: 3/4, Comparability: 2/2, Outcome: 3/3)

- • Selection: Representative of NICHD NRN centers; prospective registry; minus 1 star for survival bias
- • Comparability: Adjusted for GA, birth weight, sex, race, and major neonatal morbidities
- • Outcome: Standardized neurologic examination with GMFCS; follow-up at 18-26 months; 89.6% follow-up

## Haga et al. 2025 (Ref 8)

**Total NOS Score: 8/9** (Selection: 3/4, Comparability: 2/2, Outcome: 3/3)

- • Selection: Single tertiary center in Japan; secure medical records; outcome assessed at 3 years
- • Comparability: Adjusted for GA, birth weight, and key neonatal factors
- • Outcome: Standardized assessment using KSPD; 82% follow-up rate; adequate duration

## Yang et al. 2025 (Ref 31)

**Total NOS Score: 8/9** (Selection: 3/4, Comparability: 2/2, Outcome: 3/3)

- • Selection: Representative national cohort (KNN covers ~80% of VLBW infants in South Korea); prospective data collection with secure records; outcome not present at baseline; minus 1 star for low follow-up rate (15.7% completed BSID-III) with potential selection bias
- • Comparability: Comprehensive adjustment for GA, birth weight, head circumference, major neonatal morbidities (BPD, IVH≥3, PVL, sepsis, NEC), maternal diabetes, hypertension, and chorioamnionitis; stratified analysis by GA groups
- • Outcome: Standardized BSID-III assessment; adequate follow-up duration (18-24 months corrected age); complete assessment for those included

# GRADE Summary of Findings Table

**Outcome:** Risk of cerebral palsy in males versus females (risk ratio)

**Population:** Live-born infants across all gestational ages

**Setting:** Population-based cohorts/registries in high-income countries

# Supplementary Table 2 — GRADE Summary of Findings

*Outcome: Risk of cerebral palsy (CP) in males versus females (risk ratio) | Population: Live-born infants across gestational ages | Study design: Observational cohort studies assessed by Newcastle-Ottawa Scale (NOS)*

**Starting certainty: LOW (observational cohort evidence). Upgrading applied where formal GRADE criteria met (see footnotes). GRADE domains assessed: risk of bias, inconsistency, indirectness, imprecision, publication bias.**

**Certainty colour key:** ■ HIGH ■ MODERATE ■ LOW ■ VERY LOW

| **Outcome / Stratum (contributing studies and NOS scores)** | **Studies (comparisons)** | **Effect estimate (RR, 95% CI)** | **Risk of bias** | **Incon- sistency** | **Indirect- ness** | **Impre- cision** | **Pub- lication bias** | **Certainty (GRADE)** | **Interpretation and upgrade/downgrade rationale** |
| --- | --- | --- | --- | --- | --- | --- | --- | --- | --- |
| **Overall (all GA)** | 22 studies, 47 comparisons NOS range: 7–9/9 (median 8/9) | RR = 1.22 (95% CI 1.17–1.28) I² = 35.8% | Not serious All NOS 7–9 No selection/ measurement bias | Not serious Consistent direction all 47 comparisons; no stratum crosses null | Not serious Direct sex- specific CP measurement in same cohorts | Not serious CI 1.17–1.28 entirely above null; large N | Not serious Sex is standard registry output; no selective reporting likely | **HIGH**  ⊕⊕⊕⊕ | Males have consistently higher CP risk across all gestational ages. Upgrading applied: consistency across 47 independent comparisons + plausible confounders (IVH, PVL, NEC more prevalent in males) would attenuate rather than inflate the observed association. |
| **Very preterm (≤31 weeks)** | 13 studies, 24 comparisons NOS 9/9: Moster, Kuban, Cortese NOS 8/9: Tran, Hintz, Moore, Beaino, Kent, Neubauer, Hirvonen, Serenius, Pierrat, DeMauro | RR = 1.27 (95% CI 1.16–1.39) I² = 34.0% | Not serious Predominantly complete national registries and prospective cohorts; NOS 8–9/9 | Not serious Consistent direction all 24 comparisons; I²=34% (magnitude only) | Not serious Direct CP outcome; SCPE-based diagnosis; follow-up 2–5 years | Not serious CI 1.16–1.38 entirely above null; adequate precision | Not serious Large registry studies; no asymmetry detected | **HIGH**  ⊕⊕⊕⊕ | Consistent and precise male excess in the highest-risk group. Additional upgrade consideration: survival-mediated bias in recent cohorts (enrichment with high-risk male survivors) would attenuate the true sex difference, making RR 1.27 a conservative estimate. |
| **Moderate-to-late preterm (32–36 weeks)** | 4 studies, 8 comparisons NOS 8/9: Reid, Jöud, Larsen, Haga | RR = 1.12 (95% CI 1.00–1.26) I² = 0% | Not serious All NOS 8/9; validated registries | Not serious I²=0%; consistent direction; 4 independent populations | Not serious Direct CP measurement | SERIOUS ↓ Lower CI boundary=1.00 (reaches null); small absolute risk difference; clinical importance uncertain | Not serious | **LOW**  ⊕⊕⊝⊝ | Possible male excess but statistically uncertain: lower CI boundary reaches null. Downgraded one level for imprecision despite perfect consistency. High NOS scores do not override the mandatory imprecision downgrade when CI includes 1.00. |
| **Term (≥37 weeks)** | 6 studies, 14 comparisons NOS 8/9: Wu, Johnson, Reid, Jöud, Yang NOS 7/9: Trønnes | RR = 1.25 (95% CI 1.18–1.32) I² = 37.8% | Not serious Mostly NOS 8/9; one study 7/9 (Trønnes: mild CP under- ascertain- ment) | Not serious Consistent direction; I²=37.8% (magnitude only) | Not serious Direct CP; large population >2 million term births | Not serious CI 1.18–1.32; narrow and entirely above null | Not serious | **HIGH**  ⊕⊕⊕⊕ | Clear and precise male excess at term. Upgrading applied for consistency and precision; large underlying population provides excellent statistical power and generalisability. |
| **GA interaction (meta-regression across strata)** | 15 studies, 41 comparisons NOS range 7–9/9 | No significant interaction p = 0.863 (male excess does not vary significantly by GA) | Not serious High NOS; well-adjusted studies | Not serious Stable RRs across all strata; consistent direction | Serious ↓ Meta-regression uses aggregate study-level data; ecological bias risk | Not serious Narrow inter- stratum differences; stable across sensitivity analyses | Not serious | **MODERATE**  ⊕⊕⊕⊝ | Moderate-certainty evidence that the male excess does not vary significantly by gestational age. Upgrading applied for consistency; one downgrade retained for the ecological nature of meta-regression and limited study-level observations for the interaction test. |
| **Temporal moderation of male excess Preterm (<37 wk) (midpoint year + cohort duration)** | 32 comparisons from preterm studies NOS range 7–9/9 | β(duration)=−0.010 robust p=0.001 β(midpoint)=−0.007 CR2 p=0.268 Q_M(3)=4.26 p=0.013 R²=65.2% | Serious ↓ Both temporal variables are study-level; eco-logical inference; individual-level trends not directly testable | Not serious Both variables negative and concordant in preterm strata (predicted direction) | Serious ↓ Study-level moderators as proxies for individual-level temporal trends; causal chain not fully traceable | Serious ↓ β(midpoint) not significant by CR2 (p=0.268); k=32 study- level obs. for 2-predictor model | Not serious Temporal variables pre-specified a priori | **LOW**  ⊕⊕⊝⊝ | Low-certainty evidence that temporal cohort characteristics jointly modulate the male excess in preterm births. Start LOW; +1 internal concordance (both variables negative, joint QM p=0.013); −1 risk of bias (ecological); −1 indirectness (study-level proxies); −1 imprecision (β[midpoint] fragile). Net: LOW. |
| **Temporal moderation of male excess Very preterm (≤31 wk) (midpoint year + cohort duration) ★ Dose-response upgrade applied** | 24 comparisons from ≤31-week studies NOS 8–9/9 (biologically homogeneous population; predominantly complete national registries) | β(duration)=−0.0095 robust p=0.006 CR2 p=0.027 β(midpoint)=−0.0070 Q_M(3)=3.67 p=0.030 R²=65.6% | Serious ↓ Ecological inference applies; however homogeneous population and complete registries reduce residual bias | Not serious Both variables negative; β(duration) robust to all three inference methods; R²=65.6% | Serious ↓ (but less severe than preterm stratum): biologically homogeneous; mechanism (IVH/PVL prevention) consistent; external evidence supports hypothesis | Not serious (joint model) Joint QM p=0.030; R²=65.6%; β(duration) CR2 p=0.027. Partitioning between variables imprecise but joint effect robust | Not serious | **MODERATE**  ⊕⊕⊕⊝ | MODERATE certainty that temporal cohort characteristics modulate the male excess specifically in very preterm births. Start LOW; +1 concordance; −1 risk of bias; −1 indirectness; +1 DOSE-RESPONSE GRADIENT (effect strongest ≤31w → present <37w → absent at term; precisely predicted biological gradient). Net: MODERATE. |

**Footnotes**

1. NOS = Newcastle-Ottawa Scale (0–9); higher scores indicate higher methodological quality. All included studies scored 7–9/9; no study was rated low quality.

2. GRADE certainty symbols: ⊕⊕⊕⊕ High | ⊕⊕⊕⊝ Moderate | ⊕⊕⊝⊝ Low | ⊕⊝⊝⊝ Very Low.

3. Starting certainty: LOW for all outcomes (observational cohort study design). Upgrading applied where formal GRADE criteria were met: (1) consistency of direction across all independent comparisons with no stratum crossing the null; (2) plausible residual confounders (male-specific neonatal comorbidities: IVH, PVL, NEC, BPD) would attenuate rather than inflate the observed sex difference; (3) dose-response gradient across gestational age strata (very preterm temporal moderation only).

4. High NOS scores informed the risk of bias domain (no downgrade applied) but NOS scores alone do not constitute a GRADE upgrade criterion.

5. Imprecision downgrade for 32–36 week stratum: lower CI boundary = 1.00, reaching the null; this downgrade is mandatory regardless of study quality or consistency.

6. Temporal meta-regression: cohort midpoint year and cohort duration were used as joint moderators (Model M3: log[RR] ~ midpoint_c + duration_c, REML with Knapp-Hartung correction). Both variables are study-level ecological characteristics; individual-level temporal trends cannot be directly tested with aggregate data.

7. ★ Dose-response upgrade (very preterm temporal moderation): the temporal moderation effect exhibited a biological gradient across strata — strongest in ≤31 weeks (R²=65.6%, QM p=0.030), present in <37 weeks, absent in term births (QM p=0.763) — consistent with the hypothesis that NICU-based improvements in prevention of IVH and PVL have progressively narrowed the male-specific CP disadvantage preferentially in the most immature infants.

8. CP = cerebral palsy; GA = gestational age; IVH = intraventricular haemorrhage; PVL = periventricular leukomalacia; NEC = necrotising enterocolitis; BPD = bronchopulmonary dysplasia; RR = risk ratio; CI = confidence interval; QM = omnibus Wald test of moderators; R² = pseudo-R² (proportion of between-study variance explained by meta-regression model; approximate for multilevel models).
