# Supplementary material for: Born at higher risk: persistent male excess risk of cerebral palsy from extreme prematurity to term: a systematic review and meta-analysis
Source: Biol Sex Differ. 2026 Jun 1;17:143. doi: 10.1186/s13293-026-00935-4 (PMC13445702; doi:10.1186/s13293-026-00935-4)
Supplement: Supplementary file 3 [file 13293_2026_935_MOESM3_ESM.docx]

Suppl.3A. : Male excess risk of cerebral palsy (CP) in preterm vs term births

Suppl 3B: Male excess risk according to the midpoint year and duration of studies. (Omnibus Wald test for overall joint significance= 4.36,df=2, p=0.11). Stratified preterm and term analysis reported in graph

Suppl 3C: Male excess risk according according to the midpoint year and duration of studies stratified by very preterm, moderately preterm and term. (Omnibus Wald test for joint significance = 7.36, df=2, p=0.025)

Suppl. 3D. Funnel plot of male excess risk of cerebral palsy

Suppl. 3E: TRIM and FILL adjustment.

SUMMARY OF ADJUSTMENTS (Sensitivity Analysis) =

Category	`Original RR` `Original 95% CI` `Adjusted RR` `Adjusted 95% CI` `Studies Imputed` `Change %`

1 ≤31 weeks	1.27	[1.16 - 1.39]	1.16	[1.05 - 1.27]	8 -8.9%

2 32-36 weeks 1.12	[1.00 - 1.26]	1.10	[0.98 - 1.23]	1-1.9%

3 ≥37 weeks	1.25	[1.18 - 1.32]	1.30	[1.23 - 1.38]	5-4.7%

Suppl. 3F: Leave-one-out analysis
